# The behaviour of random forest permutation-based variable importance measures under predictor correlation

**DOI:** 10.1186/1471-2105-11-110

**Published:** 2010-02-27

**Authors:** Kristin K Nicodemus, James D Malley, Carolin Strobl, Andreas Ziegler

**Affiliations:** 1 Statistical Genetics, Wellcome Trust Centre for Human Genetics, University of Oxford, Roosevelt Drive, Oxford OX3 7BN, UK; 2 Department of Clinical Pharmacology, University of Oxford, Old Road Campus Research Building, Off Roosevelt Drive, Oxford OX3 7DQ, UK; 3 Genes, Cognition and Psychosis Program, Intramural Research Program, National Institute of Mental Health, National Institutes of Health, Room 4S-235, 10 Center Drive, Bethesda, Maryland, 20892, USA; 4 Mathematical and Statistical Computing Laboratory, Division of Computational Bioscience, Center for Information Technology, National Institutes of Health, Bethesda, Maryland, 20892, USA; 5 Department für Statistik, Ludwig-Maximilians Universität München, Ludwigstr. 33, 80539 München, Germany; 6 Institut für Medizinische Biometrie und Statistik, Universität zu Lübeck, Universitätsklinikum Schleswig-Holstein, Campus Lübeck, Maria-Goeppert-Str 1, 23562, Lübeck, Germany

## Abstract

**Background:**

Random forests (RF) have been increasingly used in applications such as genome-wide association and microarray studies where predictor correlation is frequently observed. Recent works on permutation-based variable importance measures (VIMs) used in RF have come to apparently contradictory conclusions. We present an extended simulation study to synthesize results.

**Results:**

In the case when both predictor correlation was present and predictors were associated with the outcome (H_A_), the unconditional RF VIM attributed a higher share of importance to correlated predictors, while under the null hypothesis that no predictors are associated with the outcome (H_0_) the unconditional RF VIM was unbiased. Conditional VIMs showed a decrease in VIM values for correlated predictors versus the unconditional VIMs under H_A _and was unbiased under H_0_. Scaled VIMs were clearly biased under H_A _and H_0_.

**Conclusions:**

Unconditional unscaled VIMs are a computationally tractable choice for large datasets and are unbiased under the null hypothesis. Whether the observed increased VIMs for correlated predictors may be considered a "bias" - because they do not directly reflect the coefficients in the generating model - or if it is a beneficial attribute of these VIMs is dependent on the application. For example, in genetic association studies, where correlation between markers may help to localize the functionally relevant variant, the increased importance of correlated predictors may be an advantage. On the other hand, we show examples where this increased importance may result in spurious signals.

## Background

Random forest (RF) [1] and related methods such as conditional inference forest (CIF) [2] are both tree-building methods that have been found increasingly successful in bioinformatics applications. This is especially true in statistical genetics, microarray analysis and the broad and rapidly expanding area of -omics studies. However, these types of bioinformatics applications often exhibit complex patterns of within-predictor correlation. Two recent reports have examined the impact of within-predictor correlation on RF [3,4] and have arrived at apparently divergent conclusions. Both examined whether bias in variable selection during tree-building led to biased variable importance measures (VIMs) when predictors were correlated. Strobl et al. [3] showed larger VIMs for correlated predictors under H_A _using CIF was due to the preference of correlated predictors in early splits of the trees and the particular permutation scheme employed in the computation of the permutation-based VIM. They proposed a new conditional permutation-based VIM to circumvent this inflation. In contrast, Nicodemus and Malley [4] reported that RF prefers uncorrelated predictors over all splits performed in building all trees in the forest under H_0 _and the alternative hypothesis H_A _(unless the effect size is large, e.g., an odds ratio of 5.0) because the splitting rule is based on the Gini Index. They further reported that, under H_0, _unconditional permutation-based VIMs are unbiased under within-predictor correlation for both RF and CIF, although Gini Index-based VIMs in RF are biased. In a third study, Meng et al. [5] suggest a revised tree-building algorithm and VIM that suppress the inclusion of correlated predictors in the same tree. Their findings indicate that the stronger the association with the response, the stronger the effect predictor correlation has on the performance of RF, which is in accordance to the findings of [3] and [4] with zero to high effects on the response respectively. While the identification of only those predictors associated with the response was found to be aggravated in the presence of predictor correlation, the identification of sets of predictors both associated with the response and correlated with other predictors might be useful, e.g., in genome-wide association studies, where strong LD may be present between physically proximal genetic markers.

To study the reported phenomenon in more detail and to synthesize the results of the previous studies [3-5], we conducted a simulation study using a simple linear model containing twelve predictors as first studied in [3]. This model contained a set of four strongly correlated predictors (*r *= 0.9) and eight uncorrelated predictors (*r *= 0) (Table 1). Three of the four correlated predictors and three of the eight uncorrelated predictors had non-zero coefficients. We examined the potential bias in RF and CIF during variable selection for the first splitting variable and across all the trees in the forest. We studied the impact of correlated predictors on the resulting variable importance measures generated by the two algorithms, including unscaled, unconditional permutation-based VIMs (RF and CIF), scaled permutation-based VIMs (RF) and conditional permutation-based VIMs (CIF).

**Table 1:** Bias and 95% coverage for full and single predictor models.

Predictor	True Value	Member of Correlated Group	Full Model: Bias	Full Model: 95% Coverage	Single Model: Bias	Single Model: 95% Coverage
*x*_*1*_	5	T	9.16e-04	94.6	6.31	0.0
*x*_*2*_	5	T	-6.09e-04	97.2	6.31	0.0
*x*_*3*_	2	T	-0.0014	94.6	9.02	0.0
*x*_*4*_	0	T	0.0011	96.2	10.81	0.0
*x*_*5*_	-5	F	6.77e-04	96.0	-0.015	94.2
*x*_*6*_	-5	F	2.75e-04	95.2	-0.017	94.2
*x*_*7*_	-2	F	-3.90e-04	93.8	-0.0017	95.8
*x*_*8*_	0	F	-5.26e-04	95.8	0.0050	93.4
*x*_*9*_	0	F	1.86e-04	94.6	-0.012	94.6
*x*_*10*_	0	F	2.47e-04	94.4	0.012	95.8
*x*_*11*_	0	F	-3.60e-04	95.2	0.0096	94.4
*x*_*12*_	0	F	-3.56e-06	95.8	0.0040	95.8

## Results and Discussion

In what follows, the results of RF VIM and estimated coefficients of bivariate and multiple linear regression models are compared to the coefficients that were used to generate the data by means of a multiple linear regression model. Under the alternative some predictors were influential with the coefficients reported in the methods section. Under the null hypothesis all predictors had a coefficient of zero, but the same block correlation structure.

The results are reported in terms of bias in the statistical sense that an estimator is unbiased if, on average, it reproduces the coefficient of the original model used for generating the data, and is biased if it systematically deviates from the original coefficient. However, a key point is whether or not machine learning algorithms, which are by their very nature often nonlinear and nonparametric, should be expected to approximate the linear generating model, or if they contain additional information that may be exploited in the search for highly influential predictors.

### Predictor selection frequencies

**Figure 1:**
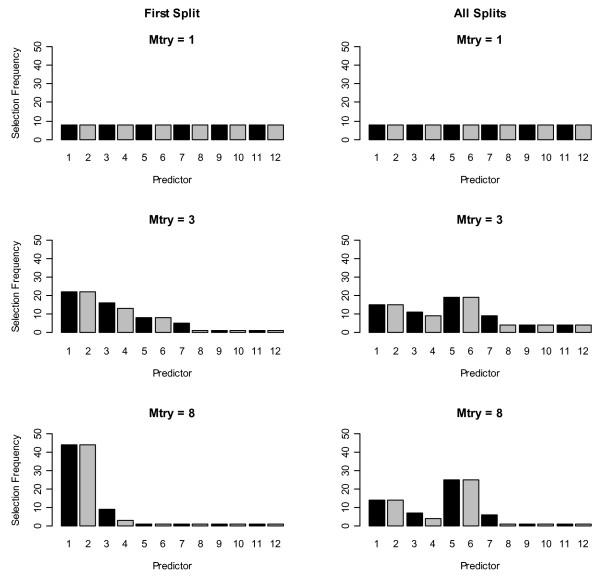
**Histograms of selection frequencies using random forest**. First row: number of variables selected at each split (mtry) = 1, 2^nd ^row: mtry = 3, 3^rd ^row: mtry = 8. First column shows the first split selection frequencies and 2^nd ^column shows the selection frequencies across all trees in all forests.

**Figure 2:**
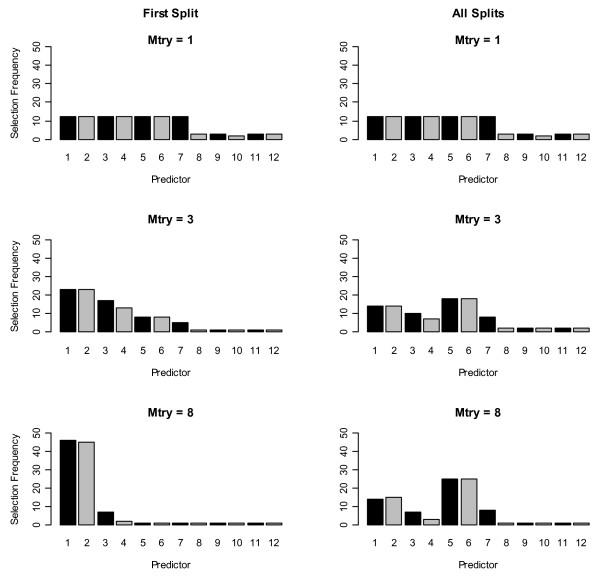
**Histograms of selection frequencies for each variable using conditional inference forest**. First row: number of variables selected at each split (mtry) = 1, 2^nd ^row: mtry = 3, 3^rd ^row: mtry = 8. First column shows the first split selection frequencies and 2^nd ^column shows the selection frequencies across all trees in all forests.

Under H_A_, correlated predictors were selected more frequently at the first split in trees grown with subsets of three and eight (mtry) randomly preselected predictors (first column of Figures 1 and 2), as reported in [3]. This was true for both algorithms. When one variable was selected for splitting (mtry = 1), CIF selected the four correlated predictors and the three uncorrelated predictors with non-zero coefficients more frequently than the uncorrelated predictors with coefficients = 0. This suggests the p-value splitting criterion in CIF is more sensitive to predictor association with the outcome than the Gini Index used by RF when mtry = 1, because CIF selected the associated predictors (or predictors strongly correlated with associated predictors) more frequently than RF at both the first split and across all splits in the forest (first row, Figures 1 and 2).

However, the pattern of selection frequencies using all splits showed the same pattern as reported in [4] for both algorithms (second column of Figures 1 and 2). Uncorrelated strongly-associated predictors (*x*_*5 *_and *x*_*6*_) were selected slightly more frequently across all trees when the pool of potential predictor variables (mtry) was set to greater than one because of competition between correlated predictors for selection into a tree (second and third rows, second column, Figures 1 and 2). Differences from results in [3] are due to different choices in tuning parameters regulating the depth of trees, as further discussed below.

As the number of variables to select from for splitting increased (i.e., as mtry went from 3 to 8) this preference for uncorrelated predictors was stronger, because the likelihood of more than one correlated predictor being in the pool of available predictors to choose from was greater. Further, we observed this same preference for uncorrelated predictors in the first split and across all splits in the tree under H_0 _for both algorithms (Additional files 1 and 2). In summary, the preference for correlated variables as the first splitting predictor was only induced when the correlated predictors were associated with the outcome. In the next section we explain why this phenomenon was observed here and in [3].

As previously reported [3], we found both algorithms showed increased selection of *x*_*4*_, which had a coefficient of 0 in the generating model, versus the uncorrelated predictors with coefficients of 0 (*x*_*8*_... *x*_*12*_). Here we give a more detailed illustration and explanation of this effect. Tree-building uses predictors sequentially in each tree and single predictors that are strongly correlated with the outcome are more likely to be selected at the first split. Statistically speaking, in the first split of each tree the split criterion is a measure of the bivariate effect of each predictor on the response, or of the marginal correlation between that predictor and the response. In the case of predictors that are strongly correlated with influential predictors, this univariate or marginal effect is almost as strong as that of the originally influential predictor. In this case, as we show in detail, even though the predictor *x*_*4 *_has a coefficient of 0 in the generating model, the correlation between *x*_*4 *_and the outcome is ~0.79 because of the correlation between *x*_*4 *_and the correlated predictors (*x*_*1*_, *x*_*2*_, and *x*_*3*_) which have non-zero coefficients.

Thus, not only the first split of a tree, but also a bivariate linear regression model indicated that predictors that were strongly correlated with an influential predictor - and can thus serve as a proxy for it - is predictive of the response. For example, within the context of a genetic association study, let us suppose there are two SNPs: SNP1 and SNP2. SNP 1 is the true causal variant associated with the outcome, which may be disease status or another phenotype, and SNP2 is in linkage disequilibrium with SNP1. In this example, because SNP 1 and SNP 2 are correlated with each other, either predictor, considered on its own, may look associated with the outcome.

The fact that in the first split feature selection was only bivariate or marginal is native to tree-building and is not related to properties of the data generating model. However, in the case of linear models it is particularly intuitive that single features can be highly predictive when considered in isolation, even when they appear with small or zero weights in the underlying models having many predictors. Therefore, linear models are used both as the data generating process and as a means of illustration here.

### Bias, 95% coverage and correlation between predictors and outcome under H_0 _and H_A_

RF and CIF consider predictors one at a time during tree-building. In particular, the model is a simple bivariate model at the first split containing the outcome and a single predictor. To better understand the preference for correlated predictors at the first split, we examined the bias, 95% coverage and correlation between the outcome and predictors using the full twelve-predictor model and twelve single-predictor (bivariate) regression models. We also calculated the true (i.e., mathematically-derived from the generating model equation) values of the coefficients and true correlations for bivariate models for each of the predictors and compared those values to the mean value of the bivariate coefficients and correlations observed in our simulations. We further considered a model retaining the same correlation structure but where all coefficients were set to 0 (H_0_).

As expected, when using the full (twelve predictor) linear regression model under H_A_, bias was small and centred around 0 (Table 1), indicating that the linear regression model can reproduce the original coefficients despite the high correlation, as expected since the generating model was linear. Ninety-five percent coverage ranged from 93.8% to 97.2%, and was centred around 95% for all predictors (Table 1). However, in the single predictor (or bivariate) regression models, bias was large for correlated predictors, ranging from 6.31 to 10.81 higher than the true value from the generating model (Table 1). The 95% coverage for all four correlated predictors was 0 (Table 1). This effect is known in statistics as a "spurious correlation" where a correlated predictor serves as a proxy and appears to be influential as long as the originally influential predictor is not included in the model. For uncorrelated predictors, the magnitude of the bias ranged from 0.0017 to 0.017 and the coverage ranged around the expected 95%.

These results are helpful to understand the preference for correlated variables at the first split in trees. At the first split, RF and CIF are simply a bivariate model containing the outcome and one predictor. As illustrated by means of the bivariate regression models, from this bivariate or marginal point of view the correlated predictors are actually more strongly associated with the outcome than the uncorrelated predictors. This explains why they were preferred at the first split, which we illustrate further below.

Another way to assess the first split preference of RF/CIF for the correlated predictors - especially for the correlated predictor which had a 0 coefficient in the generating model (*x*_*4*_) - is to calculate the mean bivariate or marginal correlation coefficient between each predictor and the outcome compared with the true value (Table 2). This showed again that, from the bivariate or marginal point of view, the correlated predictors (*x*_*1 *_- *x*_*4*_) were much more strongly correlated with the outcome (*r *~ 0.80) than the uncorrelated predictors with -5 coefficients (*x*_*5 *_and *x*_*6*_; *r *= -0.36) or the uncorrelated predictor with -2 coefficient (*x*_*7*_; *r *= -0.15). The observed values from our simulations were virtually identical to the true values, as expected. In addition, we calculated the true value of each coefficient using a single variable model and compared this value with the mean observed value from our simulations. These two values were also nearly identical (Table 2).

**Table 2:** True and observed values for single predictor model coefficients and correlations with outcome.

Predictor	True Bivariate Model β	Mean Observed Bivariate model β	True *r*(*x*_*i*_, *y*)	Mean Observed *r*(*x*_*i*_, *y*)
*x*_*1*_	11.30	11.31	0.82	0.82
*x*_*2*_	11.30	11.31	0.82	0.82
*x*_*3*_	11.00	11.02	0.80	0.80
*x*_*4*_	10.80	10.81	0.78	0.79
*x*_*5*_	-5.0	-5.02	-0.36	-0.36
*x*_*6*_	-5.0	-5.02	-0.36	-0.36
*x*_*7*_	-2.0	-2.002	-0.15	-0.15
*x*_*8*_	0.0	0.002	0.0	-3.6E-04
*x*_*9*_	0.0	0.005	0.0	-8.4E-04
*x*_*10*_	0.0	-0.012	0.0	-8.7E-04
*x*_*11*_	0.0	0.01	0.0	7.3E-04
*x*_*12*_	0.0	0.004	0.0	2.9E-04

Under H_0_, the magnitude of the bias for correlated predictors in the full model ranged from 0.00039 to 0.0015 and was similar in the single variable models. For uncorrelated predictors the magnitudes were also similar, and 95% coverage was appropriate for all predictors (see Additional file 3). Since the generating model did not contain an intercept, we re-calculated bias and 95% coverage using an intercept-free model which revealed similar results (Table 1 and Additional file 4).

### Unconditional and unscaled RF variable importance measures

**Figure 3:**
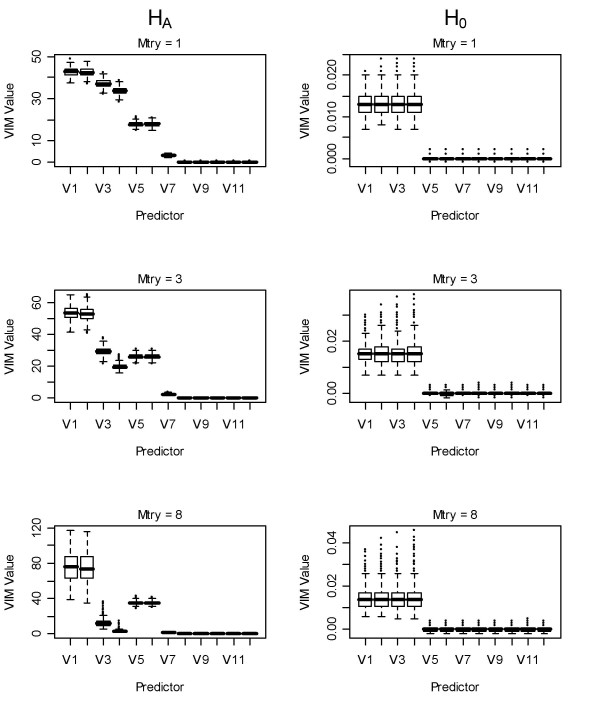
**Boxplots of unscaled permutation-based VIMs using random forest**. First column: unscaled VIMs of variables under H_A _using: 1^st ^row: mtry = 1, 2^nd ^row: mtry = 3, 3^rd ^row: mtry = 8. Second column VIMs of variables under H_0_. V = number of predictor (*x*_*i*_) in the model.

Unscaled VIMs for RF under H_A _using one variable to select from at each split (mtry = 1) showed behaviour intermediate to the bivariate models and the full regression model shown in the above section (Figure 3, column 1). Larger VIMs were observed for the correlated variables versus uncorrelated predictors, regardless of generating model coefficient, as in [3]. The correlated predictors with the largest - coefficients (*x*_*1*_, *x*_*2*_) showed larger VIMs than correlated predictors with coefficients of 2 (*x*_*3*_) or 0 (*x*_*4*_), but the latter showed higher VIMs than uncorrelated predictors with the same coefficients.

As the number of variables to select from at each split increased (mtry increased from 3 to 8), the VIMs for the correlated predictors approached the coefficients in the generating model (Figure 3, column 1). In other words, the VIMs for the correlated predictors with 2 or 0 coefficients (*x*_*3*__, _*x*_*4*_) were reduced relative to those with larger coefficients (*x*_*1*_, *x*_*2*_, *x*_*5 *_and *x*_*6*_) regardless of correlation. This is particularly true when eight variables were randomly selected at each split. The VIMs for the correlated predictors were often larger than those for the uncorrelated due to the stronger association between correlated variables and outcome when only a single correlated predictor is considered. As we showed above, this is often the case, because they are most often selected at the first split, as in [3].

To ascertain whether RF unconditional unscaled permutation-based VIMs were biased under predictor correlation under H_0_, we conducted the same analysis under H_0 _(Figure 3, column 2). This is an important aspect because a bias under the null hypothesis would mean that one would be mislead to consider some predictors that are correlated as influential, even if all predictors were random noise variables. We observed a negligible inflation in VIMs for the correlated predictors of approximately 0.014. This inflation was relatively invariant to the number of splitting variables used.

### Unconditional scaled RF variable importance measures

**Figure 4:**
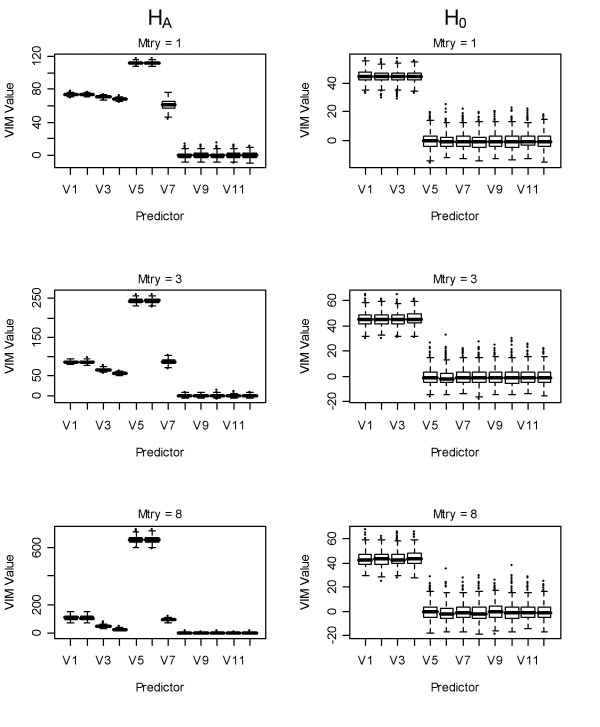
**Boxplots of scaled permutation-based VIMs using random forest**. First column: scaled VIMs of variables under H_A _using: 1^st ^row: mtry = 1, 2^nd ^row: mtry = 3, 3^rd ^row: mtry = 8. Second column VIMs of variables under H_0_. V = number of predictor (x_i_) in the model.

As previously shown [6], in the regression case the RF scaled variable importance measures grew larger with the number of trees grown (data not shown). In addition, we show here that they were also sensitive to predictor correlation (Figure 4). Under H_A_, VIMs for the uncorrelated but strongly-associated predictors (*x*_*5 *_and *x*_*6*_) were the largest because these variables were selected more frequently, thus their empirical standard error was smaller. The scaled VIMs were calculated using:

for each VIM. Under H_0_, the scaled VIMs for the correlated predictors are inflated relative to the uncorrelated predictors, which was due to the fact that the unscaled correlated predictors are always non-negative (Figure 3). Thus, the magnitude of the scaled predictors was dependent on the size of the forest [6,7] and the correlation between predictors.

### Unconditional unscaled CIF variable importance measures

**Figure 5:**
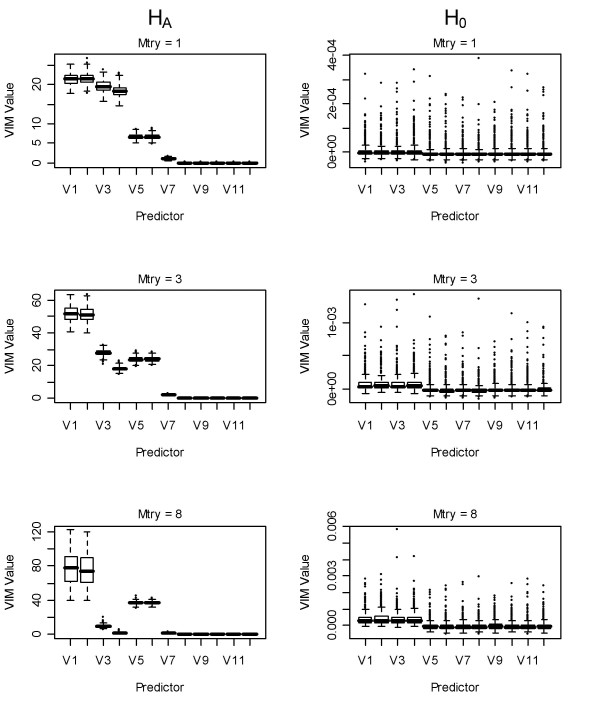
**Boxplots of unconditional permutation-based VIMs using conditional inference forest**. First column: unconditional VIMs of variables under H_A _using: 1^st ^row: mtry = 1, 2^nd ^row: mtry = 3, 3^rd ^row: mtry = 8. Second column VIMs of variables under H_0_. V = number of predictor (*x*_*i*_) in the model.

For CIF, unconditional VIMs under H_A _were very similar to those obtained using the unscaled VIM in RF (Figure 5; compare with Figure 3). Indeed, the rankings of the importance measures did not differ between the two algorithms under any value of mtry. Even the magnitude of the VIMs was similar between the two algorithms when using three or eight variables for splitting. In other words, under H_A_, the values of the unconditional VIMs from both algorithms reflected an intermediate step between single predictor linear regression models and the full twelve-predictor linear regression model, with the values for the correlated associated variables larger than for the uncorrelated associated ones.

Under H_0_, CIF showed an even smaller bias in inflation of VIMs for the correlated variables. The median difference between the VIMs for correlated versus uncorrelated variables when selecting from three predictors to split on at each node was 6.54·10^-5 ^and with eight predictors it was 4.75·10^-4^. Therefore, we did not observe substantial bias in unconditional permutation-based VIMs for either algorithm under H_0_.

### Conditional unscaled CIF variable importance measures

**Figure 6:**
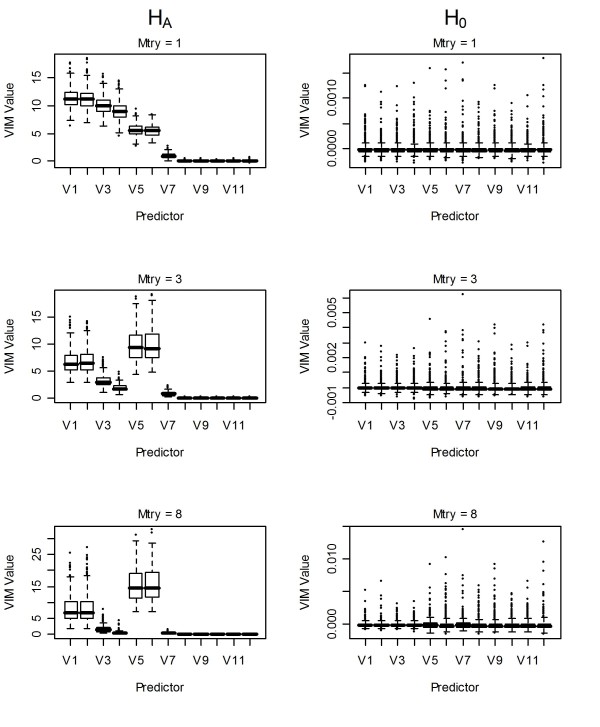
**Boxplots of conditional permutation-based VIMs using conditional inference forest**. First column: conditional VIMs of variables under H_A _using: 1^st ^row: mtry = 1, 2^nd ^row: mtry = 3, 3^rd ^row: mtry = 8. Second column VIMs of variables under H_0_. V = number of predictor (*x*_*i*_) in the model.

Using the conditional VIM from CIF and randomly selecting a single variable to split on at each node (mtry = 1) we observed a similar pattern to the unscaled unconditional VIMs from both CIF and RF (Figure 6). When the number of variables to randomly split on was larger (mtry = 3 or 8) we observed an inflation in the median VIMs for the uncorrelated strongly-associated variables (*x*_*5*_, *x*_*6*_) relative to those observed for the correlated variables that were strongly-associated (*x*_*1*_, *x*_*2*_). The inflation in the median VIMs for the uncorrelated strongly-associated variables (*x*_*5*_, *x*_*6*_*) *is contrary to the inflation found for the correlated strongly-associated variables (*x*_*1*_, *x*_*2*_) for the unconditional unscaled VIMs. However, neither pattern is consistent with the full generating model. It should be noted that the variability of the conditional VIMs for the uncorrelated strongly-associated variables (*x*_*5*_, *x*_*6*_) is very high (Figure 6), so that the inflation is less pronounced than for the correlated strongly-associated variables (*x*_*1*_, *x*_*2*_) using the unconditional VIMs. This increased variability, which was not found in [3], may be attributable to the use of larger trees in the present study and is currently the topic of further research. Under H_0_, the conditional VIMs showed a negligible bias similar to the unconditional permutation-based variable importance measure from CIF: with mtry = 3 it was 3.78·10^-5 ^and with mtry = 8 it was 1.52·10^-4^.

## Conclusions

Both algorithms, RF and CIF, more frequently selected correlated predictors at the first split in the tree, as previously reported [3]. As we showed, these predictors were more strongly associated at the bivariate or first-split level than the uncorrelated predictors associated with the outcome (here, *x*_*5 *_- *x*_*7*_). Across all splits in the forest under H_A_, CIF and RF showed variable selection frequencies relatively proportional to the effect size of the predictors. However, we observed a slight preference for selection of uncorrelated predictors as shown in [4], and this was true under both H_A _and H_0_.

Unscaled permutation-based VIMs were found to reflect an intermediate step between the bivariate and multivariate linear regression model coefficients. We did observe a very slight inflation in these VIMs for correlated variables under H_0_. However, the difference between the median VIM for correlated versus uncorrelated predictors was less than 0.014. These results highlight the importance of using Monte Carlo simulation to assess the size of observed VIMs relative to those obtained under H_0 _in applied studies. Further, the selection of the numbers of variables to randomly use at each split (mtry) should be empirically assessed when correlation between predictors is present, as we show that a large value can lead to inflation for the correlated predictors using unconditional VIMs (Figures 3 and 5) whereas the opposite was observed for the conditional VIMs (Figure 6).

Our results regarding the scaled VIM from RF agree with those of [6], who recommended the use of the unscaled VIM for regression. We also found the scaled VIM was dependent on forest size [6,7] and, as shown here, also dependent on predictor correlation. This was the only VIM showing substantial bias under H_0_.

The conditional VIM from CIF appeared to inflate the uncorrelated, strongly-associated (*x*_*5 *_and *x*_*6*_) predictor estimates of importance (both the median values and in the variability) relative to that of the correlated, strongly-associated predictors (*x*_*1 *_and *x*_*2*_). However, we found it, at present, computationally intractable for large datasets: we calculated the other VIMs on the full set of observations (n = 2,000) whereas we were only able to calculate the conditional VIM on a subset (n = 500) of observations.

One of the key aims of the study was whether the original RF VIM, whose behaviour in the presence of correlation is situated between that of bivariate and multivariate regression models, may be preferable to the conditional VIM, whose behaviour is situated closer to that of multiple regression models. The latter may be preferable in small studies where the aim is to uncover spurious correlations and identify a set of truly influential predictors among a set of correlated ones. However, in large-scale screening studies, such as genome wide association studies, the original RF VIM may be better suited to identify regions of markers containing influential predictors, because in this case correlation is usually a consequence of physical proximity of loci and thus may help localise causal variants. Since RF and CIF are nonlinear and nonparametric methods they may not be expected to exactly reproduce inferences drawn from linear regression models. In fact, we would argue that these algorithms may provide a more effective first stage information-gathering scheme than a traditional regression model. This would be especially true as the number of predictors increase, such as in the current era of genome-wide association studies or microarray studies [8].

## Methods

We investigated the behaviour of RF/CIF under predictor correlation using the same model studied in [3], which was of the form:

All predictor variables were ~*N*(0,1) and *ε *~*N*(0, 0.5). As in the original model [3], our generating model contained no intercept. The first 4 predictors (*x*_*1*_... *x*_*4*_) were block correlated at 0.9 and all other predictors were uncorrelated with the correlated predictors and one another. Predictor variables were simulated using the R package mvtnorm version 0.9-5, and MLAs were performed using the R packages randomForest version 4.5-25 [9] and party version 0.9-996. All additional analyses and simulations were conducted using R version 2.7.0 [10]. We simulated 2,000 observations per 500 replicates for all conditions and constructed forests containing 5,000 trees. Because of the computational burden in CIF, we adopted the approach of Strobl et al. [3] and calculated the conditional variable importance measure using a forest size of 500 trees and a randomly selected subset of 500 observations from each replicate.

The VIMs evaluated were all permutation-based. In RF and CIF, the unconditional VIM is calculated as the difference in mean square error (MSE) on the out-of-bag data and the MSE, also using the out-of-bag data, calculated after permuting observed values for a predictor. The differences between these two quantities are averaged across all the trees in the forest for a particular predictor. RF provides a scaled VIM where the scaling function used was:

The conditional VIM in CIF differs from the unconditional VIM by permuting one predictor within strata of sets of predictors that are correlated with that predictor [3]. RF and CIF used subsampling of 63.2% of the observations, and used a randomly-selected subset of variables for splitting (mtry) set to 1, 3 and 8 to be consistent with [3]. The minimum node size for both algorithms was set to 20, as small terminal node sizes are more sensitive to correlations between predictors [4].

We compared the frequency of variable selection in RF and CIF for both the first split and across all splits in the forest. We computed bias and 95% coverage using linear regression models for the full model containing all 12 predictors and additionally for models containing one predictor at a time. Because the generating model did not include an intercept, we calculated bias and 95% coverage using a model containing an intercept, and a model where the intercept was forced through the origin. Bias was calculated as the mean difference between the estimated coefficients for a particular predictor and the true value. Coverage was calculated as the total number of 95% confidence intervals for the estimated coefficients from the linear regression model that contained the true value, thus should be in the range of 95%. Correlation between individual predictors and outcome was calculated using Pearson's correlation coefficient. Calculating true values of the correlation between the outcome *y *and the predictors in the single-predictor models begins by letting

where *V *is the correlation matrix of the predictors and *b *is the vector of coefficients for each variable [11]. Then, the true correlations are found from

where

## Authors' contributions

KKN conceived of the study, defined the methods, performed all simulation and analysis and wrote the paper. JDM contributed to the design of the methods, wrote the paper and edited the paper. CS wrote and edited the paper. AZ edited the paper. All authors read and approved the final manuscript.

## Supplementary Material

Additional file 1 Supplementary Figure 1. Click here for file

Additional file 2 Supplementary Figure 2. Click here for file

Additional file 3 Supplementary Table 1. Click here for file

Additional file 4 Supplementary Table 2. Click here for file
