# Supplementary material for: The behaviour of random forest permutation-based variable importance measures under predictor correlation
Source: BMC Bioinformatics. 2010 Feb 27;11:110. doi: 10.1186/1471-2105-11-110 (PMC2848005; doi:10.1186/1471-2105-11-110)
Supplement: Additional file 1 — Supplementary Figure 1. [file 1471-2105-11-110-S1.PDF]

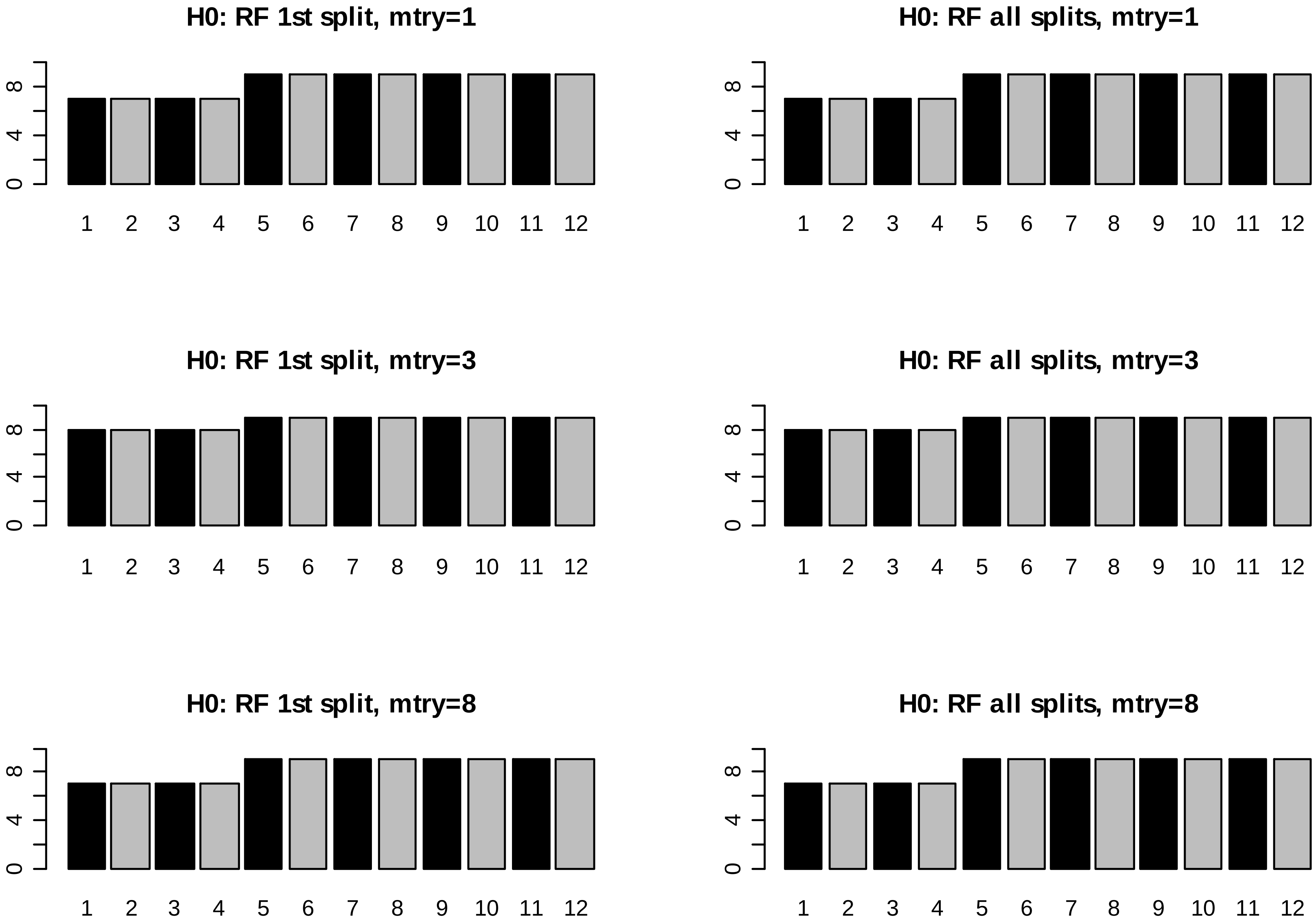
H0: RF 1st split, mtry=1
H0: RF all splits, mtry=1
H0: RF 1st split, mtry=3
H0: RF all splits, mtry=3
H0: RF 1st split, mtry=8
H0: RF all splits, mtry=8
0
4
8
1
2
3
4
5
6
7
8
9
10
11
12
