# Supplementary material for: The behaviour of random forest permutation-based variable importance measures under predictor correlation
Source: BMC Bioinformatics. 2010 Feb 27;11:110. doi: 10.1186/1471-2105-11-110 (PMC2848005; doi:10.1186/1471-2105-11-110)
Supplement: Additional file 2 — Supplementary Figure 2. [file 1471-2105-11-110-S2.PDF]

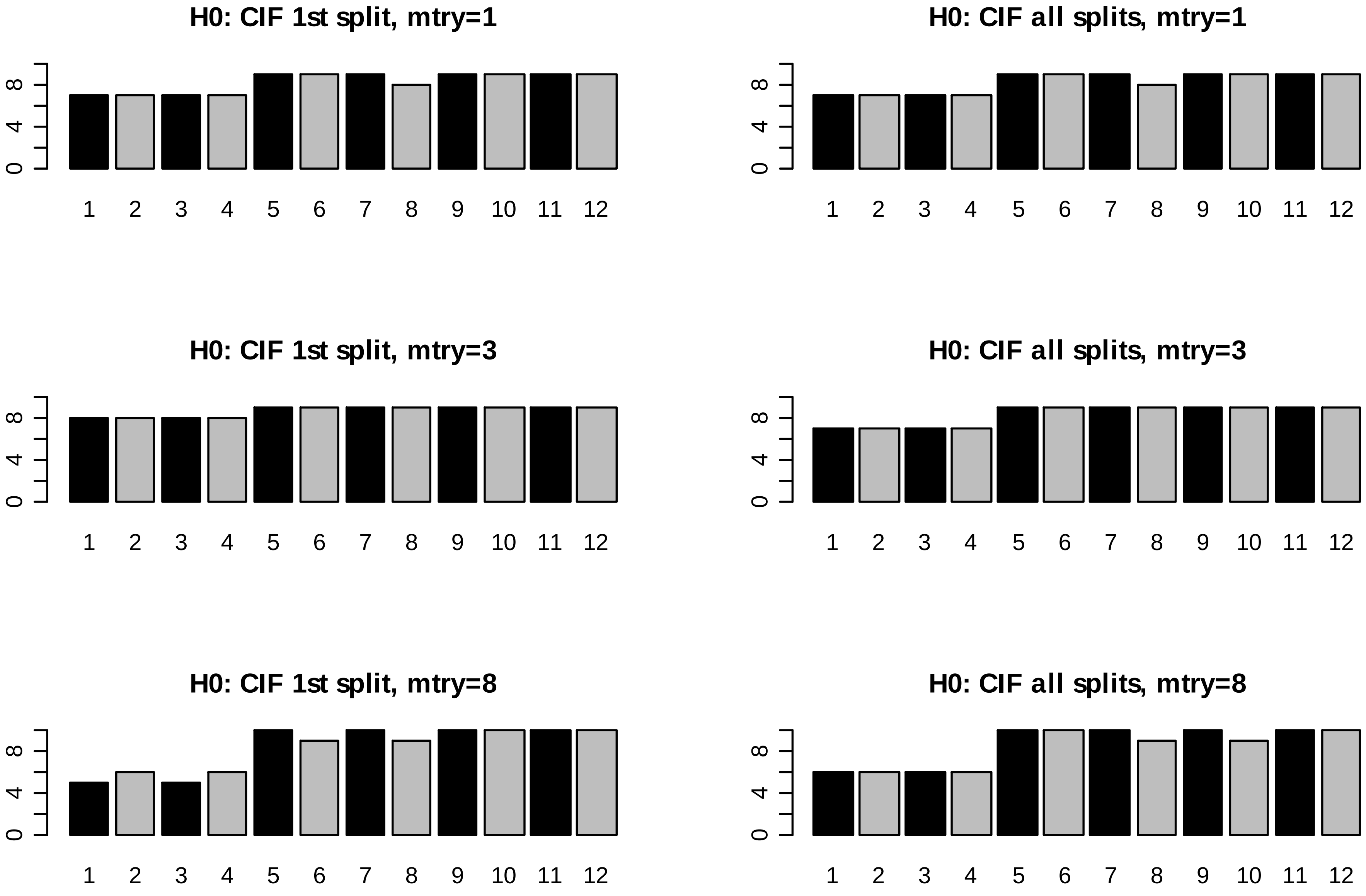
H0: CIF 1st split, mtry=1
H0: CIF all splits, mtry=1
H0: CIF 1st split, mtry=3
H0: CIF all splits, mtry=3
H0: CIF 1st split, mtry=8
H0: CIF all splits, mtry=8
0 4 8
1 2 3 4 5 6 7 8 9 10 11 12
